# ﻿First mitochondrial genome of subfamily Julodinae (Coleoptera, Buprestidae) with its phylogenetic implications

**DOI:** 10.3897/zookeys.1139.96216

**Published:** 2023-01-13

**Authors:** Zhonghua Wei, Xuyan Huang, Aimin Shi

**Affiliations:** 1 The Key Laboratory of Southwest China Wildlife Resources Conservation of the Ministry of Education, College of Life Sciences, China West Normal University, 637009, Nanchong, Sichuan Province, China China West Normal University Nanchong China; 2 College of Life Sciences, China West Normal University, 637009, Nanchong, Sichuan Province, China China West Normal University Nanchong China

**Keywords:** Jewel beetles, Julodinae, mitogenome, phylogenetics

## Abstract

Complete mitochondrial genomes of three species of the family Buprestidae were sequenced, annotated, and analyzed in this study. To explore the mitogenome features of the subfamily Julodinae and verify its phylogenetic position, the complete mitogenome of *Julodisvariolaris* was sequenced and annotated. The complete mitogenomes of *Ptosimachinensis* and *Chalcophorajaponica* were also provided for the phylogenetic analyses within Buprestidae. Compared to the known mitogenomes of Buprestidae species varied from 15,499 bp to 16,771 bp in length, three newly sequenced mitogenomes were medium length (15,759–16,227 bp). These mitogenomes were encoded 37 typical mitochondrial genes. Among the three studied mitogenomes, Leu2 (L2), Ser2 (S2), and Pro (P) were the three most frequently encoded amino acids. Within the Buprestidae, the heterogeneity in sequence divergences of Agrilinae was highest, whereas the sequence homogeneity of Chrysochroinae was highest. Moreover, phylogenetic analyses were performed based on nucleotide matrix (13 PCGs + 2 rRNAs) among the available sequenced species of Buprestidae using Bayesian Inference and Maximum Likelihood methods. The results showed that the Julodinae was closely related to the subfamily Polycestinae. Meanwhile, the genera *Melanophila*, *Dicerca*, and *Coomaniella* were included in Buprestinae, which was inconsistent with the current classification system of Buprestidae. These results could contribute to further studies on genetic diversity and phylogeny of Buprestidae.

## ﻿Introduction

The family Buprestidae is one of the largest families in Coleoptera, including six subfamilies, 521 genera, and more than 15,000 species distributed worldwide (Bellamy 2008; Kubáň et al. 2016). In this family, all species are phytophagous. The adults are feeders on flowers, leaves and stems, whereas the larvae are internal feeders in roots and stems, or feed on the foliage of woody and herbaceous plants, the larvae of Julodinae are soil habitants feeding externally by the roots (Bellamy and Volkovitsh 2016). Different groups have different functions covered ecological, social and economic functions, such as: most larvae of Buprestinae and Chrysochroinae are important decomposers of woody plants; with most species being ornamental beetles with attractive metallic luster; many species of Agrilinae are forest and agricultural pests; and some species of the tribes Stigmoderini, Acmaeoderini, and Anthaxiini are pollinator taxa. Although some buprestid taxonomists have made important contributions to the classification based on morphological analyses (Cobos 1980, 1986; Tôyama 1987; Hołyński 1988, 1993, 2009; Kolibáè 2000; Bellamy 2003), the problems of the overall classification of Buprestidae remain.

In the past two decades, the mitochondrial genome emerged as important molecular data for higher-level phylogenetic analyses (Saccone et al. 1999; Timmermans et al. 2010, 2016; Cameron 2014; Li et al. 2015; Qin et al. 2015; Nie et al. 2020, 2021; Motyka et al. 2022; Zheng et al. 2022), evolutionary strategies (Krzywinski et al. 2011; Nie et al. 2019; Motyka et al. 2022; Zhang et al. 2022), and genetic diversity analyses (Lim et al. 2021). The buprestid mitogenome also caught the attention of taxonomists. In Buprestidae, the first complete mitogenome of *Chrysochroafulgidissima* (Schönherr, 1817) was reported by Hong et al. (2009). In the same year, the mitogenome of *Acmaeodera* sp. was used to analyze the nonstationary evolution and compositional heterogeneity of Coleoptera. To date, only 22 buprestid mitogenomes (Table 1) have been reported worldwide, including three newly generated in this study.

**Table 1.** Information on the mitogenomes of Buprestidae and outgroup taxa used for phylogenetic analysis.

Subfamily	Taxa	Accession No.	Genome size (bp)	A+T%	AT-skew	Reference
Agrilinae	*Coraebusdiminutus* Gebhardt, 1928	OK189521	15,499	68.42	0.12	Wei 2022
	*Coraebuscloueti* Théry, 1895	OK189520	15,514	69.27	0.11	Wei 2022
	*Coraebuscavifrons* Descarpentries & Villiers, 1967	MK913589	15,686	69.79	0.12	Cao and Wang 2019a
	*Meliboeussinae* Obenberger, 1935	OK189522	16,108	72.42	0.11	Wei 2022
	*Sambusfemoralis* Kerremans, 1892	OK349489	15,367	73.23	0.12	Wei 2022
	*Agrilussichuanus* Jendek, 2011	OK189519	16,521	71.73	0.12	Wei 2022
	*Agrilusplanipennis* Fairmaire, 1888	KT363854	15,942	71.90	0.12	Duan et al. 2017
	*Agrilusmali* Matsumura, 1924	MN894890	16,204	74.46	0.08	Sun et al. 2020
	*Trachysauricollis* Saunders, 1873	MH638286	16,429	71.05	0.10	Xiao et al. 2019
	*Trachystroglodytiformis* Obenberger, 1918	KX087357	16,316	74.62	0.10	Unpublished
	*Trachysvariolaris* Saunders, 1873	MN178497	16,771	72.11	0.11	Cao and Wang 2019b
Buprestinae	*Melanophilaacuminata* (De Geer, 1774)	MW287594	15,853	75.66	0.02	Peng et al. 2021
	*Anthaxiachinensis* Kerremans, 1898	MW929326	15,881	73.61	0.09	Chen et al. 2021
	*Coomaniellacopipes* Jendek & Pham, 2013	OL694145	16,196	74.47	0.03	Huang et al. 2022
	*Coomanielladentata* Song, 2021	OL694144	16,179	76.59	0.01	Huang et al. 2022
Chrysochroinae	*Chrysochroafulgidissima* (Schönherr, 1817)	EU826485	15,592	69.92	0.15	Hong et al. 2009
	*Chalcophorajaponica* (Gory, 1840)	OP388437	15,759	67.97	0.13	In this study
	*Chalcophorajaponica* (Gory, 1840)	OM161962	15,759	67.94	0.13	Weng et al. 2022
	*Dicercacorrugata* Fairmaire, 1902	OL753086	16,276	71.76	0.09	Huang et al. 2022
Polycestinae	*Acmaeodera* sp.	FJ613420	16,217	68.41	0.11	Sheffield et al. 2009
	*Ptosimachinensis* Marseul, 1867	OP388449	16,115	67.00	0.13	In this study
Julodinae	*Julodisvariolaris* (Pallas, 1771)	OP390084	16,227	70.43	0.12	In this study
outgroup	*Heterocerusparallelus* Gebler, 1830	KX087297	15,845	74.03	0.13	Unpublished
	*Dryopsernesti* Gozis, 1886	KX035147	15,672	72.98	0.07	Unpublished

To date, the mitogenome of the subfamily Julodinae has not been reported. The lack of the data on complete mitogenome of Julodinae species has limited our understanding of the real phylogenetic relationships within jewel beetles. The single molecular phylogenetic analysis, including Julodinae, showed that Julodinae is monophyletic group and close to Polycestinae (Evans et al. 2015). The subfamily Julodinae includes one tribe and six genera (Hołyński 2014). The described Julodinae species are mainly distributed in the arid and semiarid zones of the Ethiopian and Palaearctic regions, except for the species of the genus *Sternocera* Eschscholtz, 1829 distributed in humid tropical zones of Asia and Africa (Bellamy 2008; Hołyński 2014).

In the present study, three complete mitogenomes are sequenced and annotated, of which that of *Julodisvariolaris* (Pallas, 1771) is the first complete mitogenome sequence to be reported in the subfamily Julodinae. In China, this species is widely distributed in Xinjiang Uygur Autonomous Region. The adults, appearing in May and June, feeder on the leaves of *Haloxylonammodendron* (Meyer, 1829) and the larvae feeder on the roots of this plant. Additionally, the complete mitogenomes of *Chalcophorajaponica* (Gory, 1840) (Chrysochroinae: Chalcophorini) and *Ptosimachinensis* Marseul, 1867 (Polycestinae: Ptosimini) are provided for phylogenetic analyses, which are also enriching the diversity of mitogenomes studied in Buprestidae. The total length of the mitogenome in *C.japonica* was consistent with the results of Weng et al. (2022). In order to explore the phylogenetic position of the subfamily Julodinae, phylogenetic analyses of the family Buprestidae were performed based on a nucleotide matrix (13 PCGs + 2 rRNAs) among buprestid species using Bayesian Inference (BI) and Maximum Likelihood (ML) methods.

## ﻿Materials and methods

### ﻿Sampling and DNA extraction

Specimens of *J.variolaris* were collected on *H.ammodendron* in the vicinities of Turpan City, Xinjiang Uygur Autonomous Region, China, on 14 May 2022. Specimens of *P.chinensis* were collected from Dayaoshan Mountains in Guangxi Zhuang Autonomous Region, China, on 20 March 2021. Specimens of *C.japonica* were collected from Quanzhou City, Fujian Province, China, on 23 February 2021. The above specimens are preserved in 95% alcohol at -24 °C in specimen collection at China West Normal University, Nanchong, China. Next-generation sequencing and assembly were performed by Beijing Aoweisen Gene Technology Co. Ltd. (Beijing, China) to obtain the complete mitogenome sequences.

### ﻿Sequence assembly, annotation, and analysis

The raw data were processed using Trimmomatic v. 0.35 (Bolger et al. 2014) to remove low-quality reads and obtain a high-quality clean data. Finally, 4.8 Gb, 5.28 Gb, and 6.8 Gb clean data were obtained to assemble complete mitogenome of *J.variolaris*, *P.chinensis*, and *C.japonica*, respectively. Three mitogenome sequences were annotated using Geneious 11.0.2 (Kearse et al. 2012) based on the invertebrate mitochondrial genetic code. All tRNA genes were reconfirmed using the online tool MITOS Web Server (Bernt et al. 2013) and the second structures were further predicted using tRNAscan-SE server v. 1.21 (Lowe and Chan 2016). Two rRNA genes were identified by alignment with other buprestid rRNA sequences. Three mitogenome maps were drawn using Organellar Genome Draw v. 1.3.1 (Greiner et al. 2019). Strand asymmetry of mitogenome sequence was calculated using the formulae reported by Perna and Kocher (1995): AT-skew = (A – T)/(A + T), and GC-skew = (G – C)/(G + C). The base composition and relative synonymous codon usage (RSCU) values of three mitogenome sequences were determined using MEGA v. 12.0.0 (Kumar et al. 2016). The non-synonymous substitutions (Ka) and synonymous substitutions (Ks) of all PCG genes were calculated using DnaSP v. 5 (Librado and Rozas 2009). The tandem repeat elements of control region (CR, also known as A + T-rich region) were detected by the online tool Tandem Repeats Finder (Benson 1999). The heterogeneous analysis of nucleotide matrix (13 PCGs + 2 tRNAs) was performed using AliGROOVE v. 1.06 (Kück et al. 2014).

### ﻿Phylogenetic analysis

To investigate mitogenome arrangement patterns in Buprestidae, the gene orders of all known buprestid mitogenomes were compared with that of closely related taxa. A total of 22 buprestid mitogenomes (Table 1), including three newly generated sequences in this study, were subjected for phylogenetic analyses, using *Heterocerusparallelus* Gebler, 1830 (Heteroceridae) and *Dryopsernesti* Gozis, 1886 (Dryopidae) as outgroups (Xiao et al. 2019; Huang et al. 2022; Wei 2022). The test of substitution saturation for the dataset (13 PCGs + 2 rRNAs) was performed with DAMBE to test whether the sequence is suitable for constructing a phylogenetic tree (Xia 2017). Then, the phylogenetic trees were reconstructed using nucleotide matrix 13 PCGs + 2 rRNAs based on ML and BI methods. The nucleotide matrix was aligned using ClustalW (Thompson et al. 1994) and trimmed by trimAl v. 1.2 (Capella-Gutiérrez et al. 2009). In BI and ML analyses, the best-fit models were deduced by ModelFinder (Kalyaanamoorthy et al. 2017). The phylogenetic trees were reconstructed using IQ-tree v. 1.6.8 (Guindon et al. 2010) and MrBayes v. 3.2.6 (Ronquist et al. 2012) integrated into PhyloSuite v. 1.2.2 (Zhang et al. 2020). During this analyzing process, PhyloSuite was run with previous parameters (Wei 2022).

## ﻿Results

### ﻿Genome organization and base composition

We sequenced and annotated the complete mitogenome of *J.variolaris* (GenBank No. OP390084), *P.chinensis* (No. OP388449), and *C.japonica* (No. OP388437). Overall, these mitogenome sequences were 15,759 to 16,227 bp in length, which are medium length in Buprestidae (Table 1). It is a circular, double-stranded ring that includes 37 insect mitochondrial genes (13 PCGs, 22 tRNAs, and 2 rRNAs) and an A + T-rich region (control region, CR).

In these three mitogenome, the N-strand encoded the sense-strand of 14 genes (*nad1*, *nad4L*, *nad4*, *nad5*, *trnQ*, *trnV*, *trnL1*, *trnP*, *trnH*, *trnF*, *trnY*, *trnC*, *rrnL*, and *rrnS*), while the J-strand encoded the sense-strand of the remaining 23 genes (Table 2), which was consistent with the known buprestid species (Cao and Wang 2019a, b; Xiao et al. 2019; Chen et al. 2021; Peng et al. 2021; Huang et al. 2022; Wei 2022; Weng et al. 2022).

**Table 2.** The three newly annotated Buprestidae mitogenomes. The order of the three species in the table is as follows: *Julodisvariolaris*, *Ptosimachinensis*, and *Chalcophorajaponica*. – not determined.

Gene	Strand	Position From	To	Start codons	Stop codons	Intergenic nucleotides
*trnI*	J	1/1/1	66/64/67			0/0/0
*trnQ*	N	64/65/65	134/133/133			-3/0/-3
*trnM*	J	134/133/133	202/201/201			-1/-1/-1
*nad2*	J	203/202/202	1228/1221/1224	ATT/ATT/ATC	TAA/TAA/TAA	0/0/0
*trnW*	J	1241/1220/1223	1306/1285/1291			12/-2/-2
*trnC*	N	1299/1278/1284	1360/1341/1345			-7/-7/-7
*trnY*	N	1361/1343/1346	1426/1406/1409			0/1/0
*cox1*	J	1428/1408/1411	2958/2941/2943	–/–/–	T(AA)/T(AA)/TAA	1/1/1
*trnL2*	J	2959/2942/2944	3024/3006/3009			0/0/0
*cox2*	J	3025/3007/3010	3709/3691/3697	ATA/ATA/ATA	T(AA)/T(AA)/T(AA)	0/0/0
*trnK*	J	3710/3692/3698	3780/3761/3767			0/0/0
*trnD*	J	3780/3762/3768	3845/3824/3829			-1/0/0
*atp8*	J	3846/3825/3887	4004/3983/4042	ATT/ATT/ATT	TAA/TAA/TAA	0/0/57
*atp6*	J	3998/3977/4036	4672/4651/4710	ATG/ATG/ATG	TAA/TAA/TAA	-6/-7/-7
*cox3*	J	4672/4651/4710	5458/5439/5496	ATG/ATG/ATG	T(AA)/TAA/T(AA)	-1/-1/-1
*trnG*	J	5459/5447/5497	5522/5512/5558			0/7/0
*nad3*	J	5523/5513/5559	5876/5866/5912	ATT/ATT/ATT	TAG/TAG/TAG	0/0/0
*trnA*	J	5875/5865/5911	5940/5929/5974			-2/-2/-2
*trnR*	J	5940/5934/5975	6006/5998/6035/			-1/4/0
*trnN*	J	6006/6002/6035	6070/6066/6099			-1/3/-1
*trnS1*	J	6071/6067/6100	6137/6131/6166			0/0/0
*trnE*	J	6138/6132/6168	6201/6197/6229			0/0/1
*trnF*	N	6201/6196/6229	6265/6260/6292			-1/-2/-1
*nad5*	N	6265/6260/6293	7983/7978/8012	ATA/ATC/GTG	TAA/TAA/T(AA)	-1/-1/0
*trnH*	N	7984/7979/8013	8047/8042/8075			0/0/0
*nad4*	N	8048/8042/8076	9380/9379/9411	ATG/ATG/ATG	T(AA)/TAA/T(AA)	0/-1/0
*nad4L*	N	9374/9373/9405	9664/9666/9695	ATG/ATG/GTG	TAA/TAA/TAA	-7/-7/-7
*trnT*	J	9667/9669/9698	9731/9733/9762			2/2/2
*trnP*	N	9731/9733/9763	9795/9798/9827			-1/-1/0
*nad6*	J	9797/9800/9829	10,303/10,306/10,335	ATA/ATA/ATC	TAA/TAA/TAA	1/1/1
*cytb*	J	10,303/10,306/10,335	11,454/11,448/11,474	ATG/ATG/ATG	TAG/TAA/TAG	-1/-1/-1
*trnS2*	J	11,453/11,447/11,473	11,519/11,512/11,539			-2/-2/-2
*nad1*	N	11,539/11,536/11559	12,489/12,480/12,509	TTG/TTG/TTG	TAA/TAA/TAG	39/33/19
*trnL1*	N	12,491/12,482/12,511	12,554/12,546/12,574			1/1/1
*rrnL*	N	12,555/12,547/12,575	13,855/13,845/13,873			0/0/0
*trnV*	N	13,856/13,846/13,847	13,925/13,915/13,943			0/0/-27
*rrnS*	N	13,926/13,916/13,944	147,17/14,664/14,679			0/0/0
A+T-rich region		14,718/14,665/14,680	16,227/16,115/15,759			0/0/0

These three mitogenome sequences had a high A + T content, with an average of 68.47%, showing a strong AT bias (Suppl. material 1: table S1). Among them, the A + T content of *J.variolaris* (70.43%) was higher than of both *C.japonica* (67.97%) and *P.chinensis* (67.00%). These three mitogenome sequences showed a positive AT skew (0.12–0.13) and negative GC skew (-0.22), which is consistent with the known buprestid species. In this study, there were 21 gaps in three mitogenome sequences, which varied from 1 bp to 57 bp. The longest intergenic spacer (bp) was located between *trnD* and *atp8* genes in *C.japonica*. There were 41 overlapping gene regions in total, ranging from 1 bp to 27 bp in length.

### ﻿Protein-coding genes, codon usage, and nucleotide diversity

In Julodinae, the concatenated length of 13 PCGs of *J.variolaris* (Julodinae) was 11,170 bp, which encoded 3715 amino acid residues. In *P.chinensis* (Polycestinae), the total length of 13 PCGs was 11,162 bp, which encoded 3710 amino acid residues. In *C.japonica* (Chrysochroinae), the total length of 13 PCGs was 11,161 bp, which encoded 3710 amino acid residues. Compared with the other known buprestid species (Chen et al. 2021; Peng et al. 2021; Huang et al. 2022; Wei 2022; Weng et al. 2022), the concatenated length of 13 PCGs and the number of amino acid-coding codons of Julodinae is slightly higher than in other subfamilies.

The majority of PCGs directly used ATN as the start codon, but the exceptions were *nad1* (*J.variolaris*, *P.chinensis*, and *C.japonica*), *nad4L* (*C.japonica*), and *nad5* (*C.japonica*) genes which started with TTG, GTG, and GTG, respectively. The unusual start codon TTG was also reported in Agrilinae (Wei 2022) and Buprestinae (Huang et al. 2022). The start codon of the *cox1* gene in these three mitogenomes was not determined, which may use non-canonical start codons (Friedrich and Muquim 2003; Fenn et al. 2007; Yang et al. 2013; Wang et al. 2021; Wu et al. 2022). There were three types of stop codons, TAA, TAG, and an incomplete stop codon T, which was completed by the addition of 3’ A residues to the mRNA.

**Figure 1.**
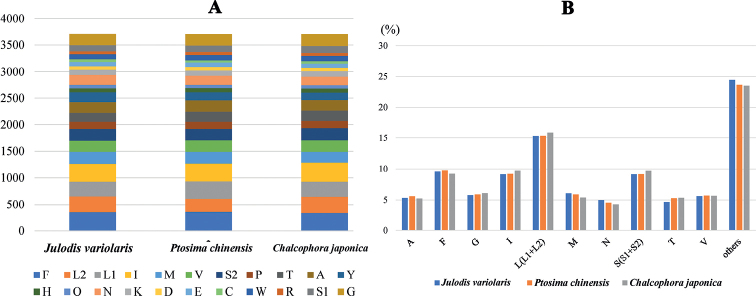
Numbers of different amino acids in the three new mitogenome sequences **A** and the percentages of the top ten amino acids **B** the stop codon is not included in these graphs.

**Figure 2.**
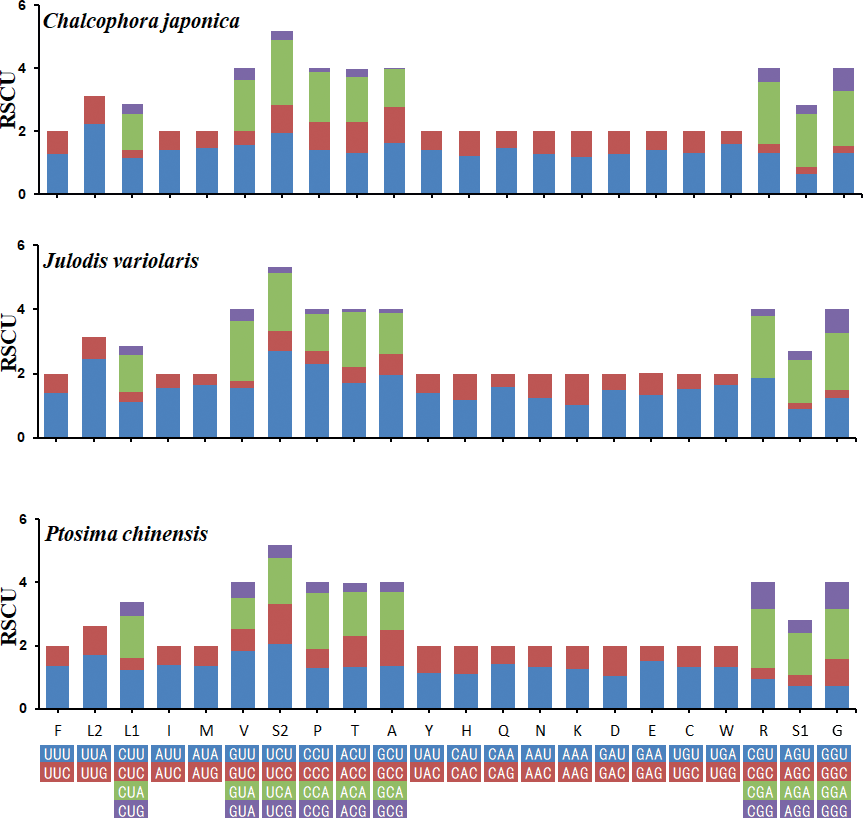
Relative synonymous codon usage (RSCU) of the three newly sequenced mitogenomes.

To investigate further, the frequency of synonymous codon usage and relative synonymous codon usage (RSCU) values were calculated and presented. Taken together, the three most frequently used amino acids were L2, S2, and P (Fig. 1A, B), and the most frequently used codons were TTA (L2), TCT (S2), and CCT (P) (Fig. 2).

**Figure 3.**
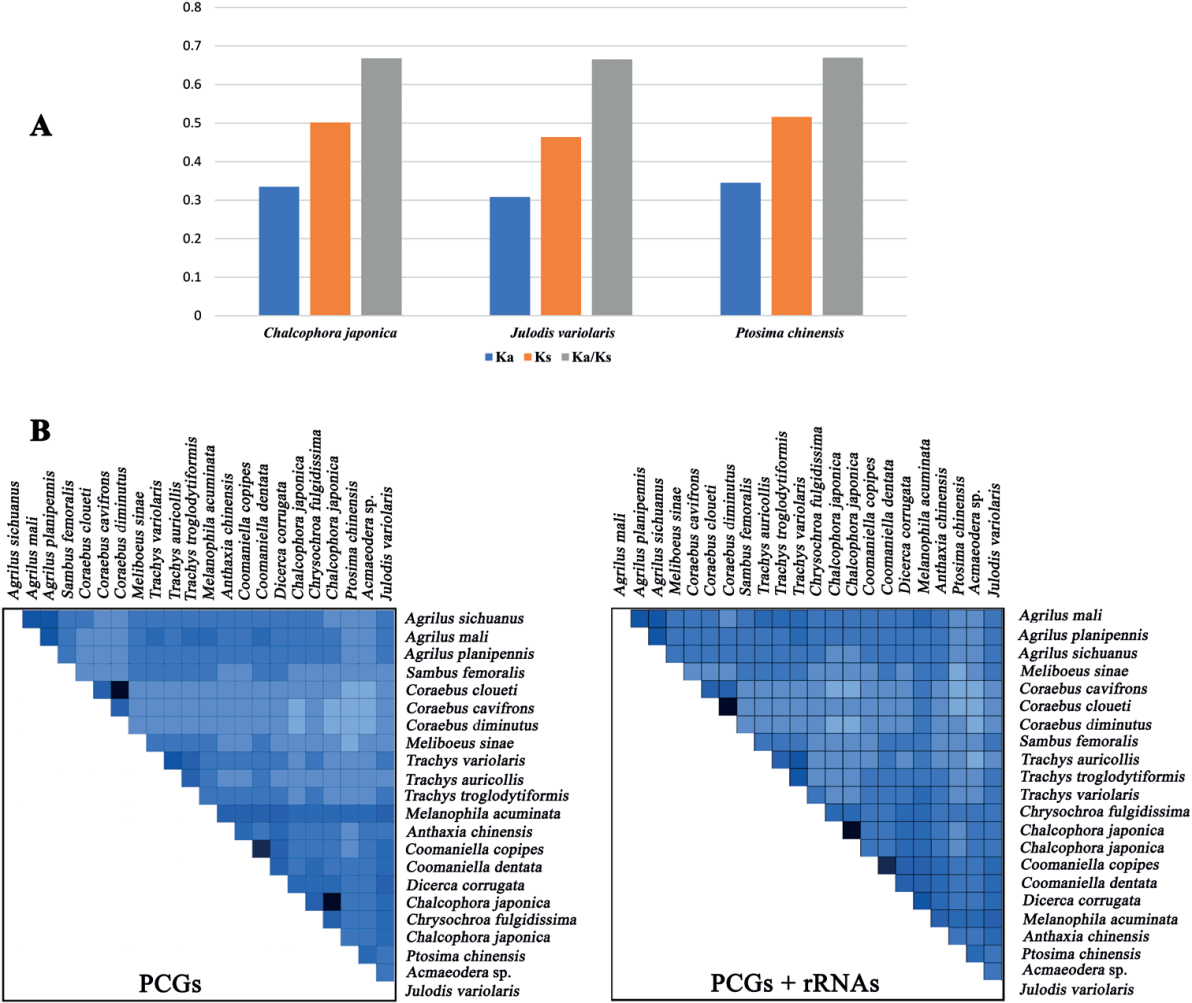
Evolutionary rates of mitochondrial genomes in three new mitogenome sequence (**A**) and the heterogeneity of two dataset in Buprestidae (**B**).

The Ka/Ks ratio can be used to estimate whether a sequence is undergoing negative, neutral, or positive selection (Hurst 2002; Mori and Matsunami 2018). The ratio of Ka/Ks for each mitogenome sequence was calculated using *Anthaxiachinensis* Kerremans, 1898 as the reference sequence (Fig. 3A). In three mitogenome sequences, values of Ka, Ks, and Ka/Ks were all less than 1, suggesting the presence of purifying selection in these three species.

### ﻿Ribosomal and transfer RNA genes, and heterogeneity

The rRNA genes were located between the A + T-rich region and *trnL1*, and separated by *trnV*, which is consistent with previous studies (Duan et al. 2017; Cao and Wang 2019a, b; Xiao et al. 2019; Sun et al. 2020; Chen et al. 2021; Peng et al. 2021; Huang et al. 2022; Wei 2022; Weng et al. 2022). The total length of rRNA genes ranged from 2035 bp (*C.japonica*) to 2093 bp (*J.variolaris*), of which the length of *16S* gene ranged from 1299 bp (*C.japonica* and *P.chinensis*) to 1301 bp (*J.variolaris*). The A + T content of rRNA genes ranged from 71.50% (*C.japonica*) to 74.30% (*J.variolaris*).

**Figure 4.**
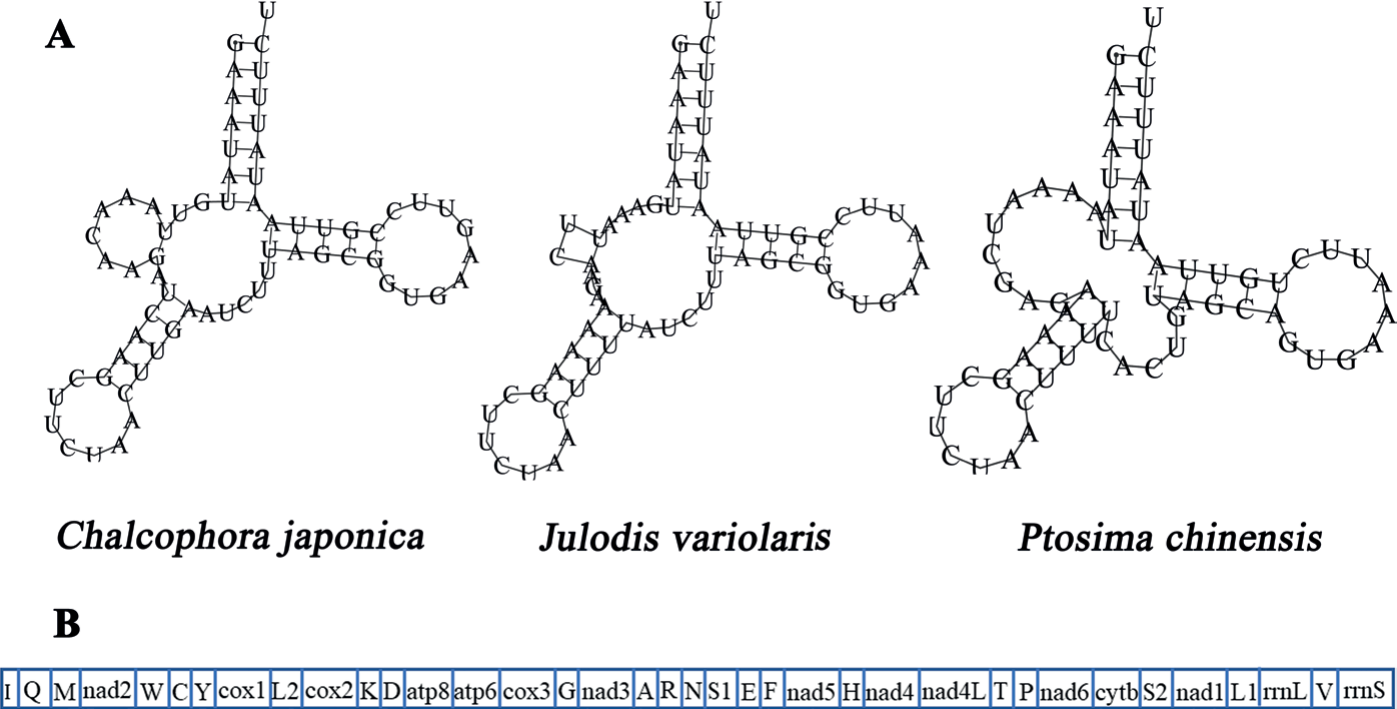
The predicted secondary cloverleaf structure for the *trnS1* of three new mitogenomes (**A**) and the gene order of known buprestid mitogenomes (**B**).

The concatenated lengths of all tRNA genes ranged from 1437 bp (*C.japonica*) to 1456 bp (*J.variolaris*), whereas individual tRNA genes ranged from 61 bp (*trnR*) to 71 bp (*trnK*), of which eight tRNA genes were encoded on the N-strand and the remaining 14 genes encoded on the J-strand. The predicted secondary structure of tRNAs showed a standard clover-leaf structure (Suppl. material 1: figs S2–S4), except for *trnS1* (Fig. 4A), which lacked the dihydrouridine arm, and formed a loop commonly found in other insects (Xiao et al. 2011; Park et al. 2012; Yu et al. 2016; Yan et al. 2017; Yu and Liang 2018; Li et al. 2019). The UG mismatches were detected in some tRNAs (Suppl. material 1: figs S2–S4), which also appeared in other buprestid species (Sun et al. 2020; Chen et al. 2021; Huang et al. 2022; Wei 2022; Weng et al. 2022).

The degree of heterogeneity of the PCGs + RNAs dataset was higher than that of the PCGs dataset (Fig. 3B). Additionally, the heterogeneity in sequence divergences was slightly stronger for Agrilinae than for other families (Fig. 3B). The heterogeneity in sequence homogeneity was higher for Chrysochroinae than other families.

### ﻿A + T-rich region and gene arrangement

The A + T-rich region was the largest non-coding region in mitogenome, located between *trnI* and *rrnS*. This region, containing regulatory elements correlated with the regulation of replication and transcription (Zhang et al. 1995), plays a very important role in molecular evolution (Zhang and Hewitt 1997). The length of A + T-rich region ranged from 1080 bp (*C.japonica*) to 1510 bp (*J.variolaris*), which are of medium length in the Buprestidae (Sun et al. 2020; Huang et al. 2022; Wei 2022). The A + T content of the A + T-rich region of *C.japonica* (75.93%) and *P.chinensis* (78.38%) was found to be higher than that of the whole genome (67.97%, 67.00%), PCGs (66.46%, 64.55%), rRNAs (71.50%, 72.51%), and tRNAs (68.82%, 71.46%), whereas the A + T content of *J.variolaris* (72.85%) was lower than that of whole genome (70.43%), PCGs (68.82%), rRNAs (74.30%), and tRNAs (74.79%).

The tandem repeat regions of three species were detected in this study. The repeat regions in each of the three new mitogenomes differ from each other in length and copy number of tandem repeat units. The repeat region of *J.variolaris* was 43 bp in length, comprising a 17 bp and a 26 bp tandem repeat element. In contrast, in *P.chinensis*, the total length of the repeat sequence was 111 bp, consisting of three incomplete repeat units. These tandem repeat elements are slightly shorter than those of Agrilinae (Wei 2022).

The gene rearrangements were regarded as important molecular markers for exploring the evolution and phylogeny of insects (Dowton et al. 2002; Cameron 2014). All the buprestid mitogenomes released in GenBank were compared and analyzed, with one mitogenome arrangement pattern exhibited in Buprestidae (Fig. 4B). The mitochondrial gene order of these three species was consistent with other known buprestid mitogenomes.

### ﻿Phylogenetic analysis

**Figure 5.**
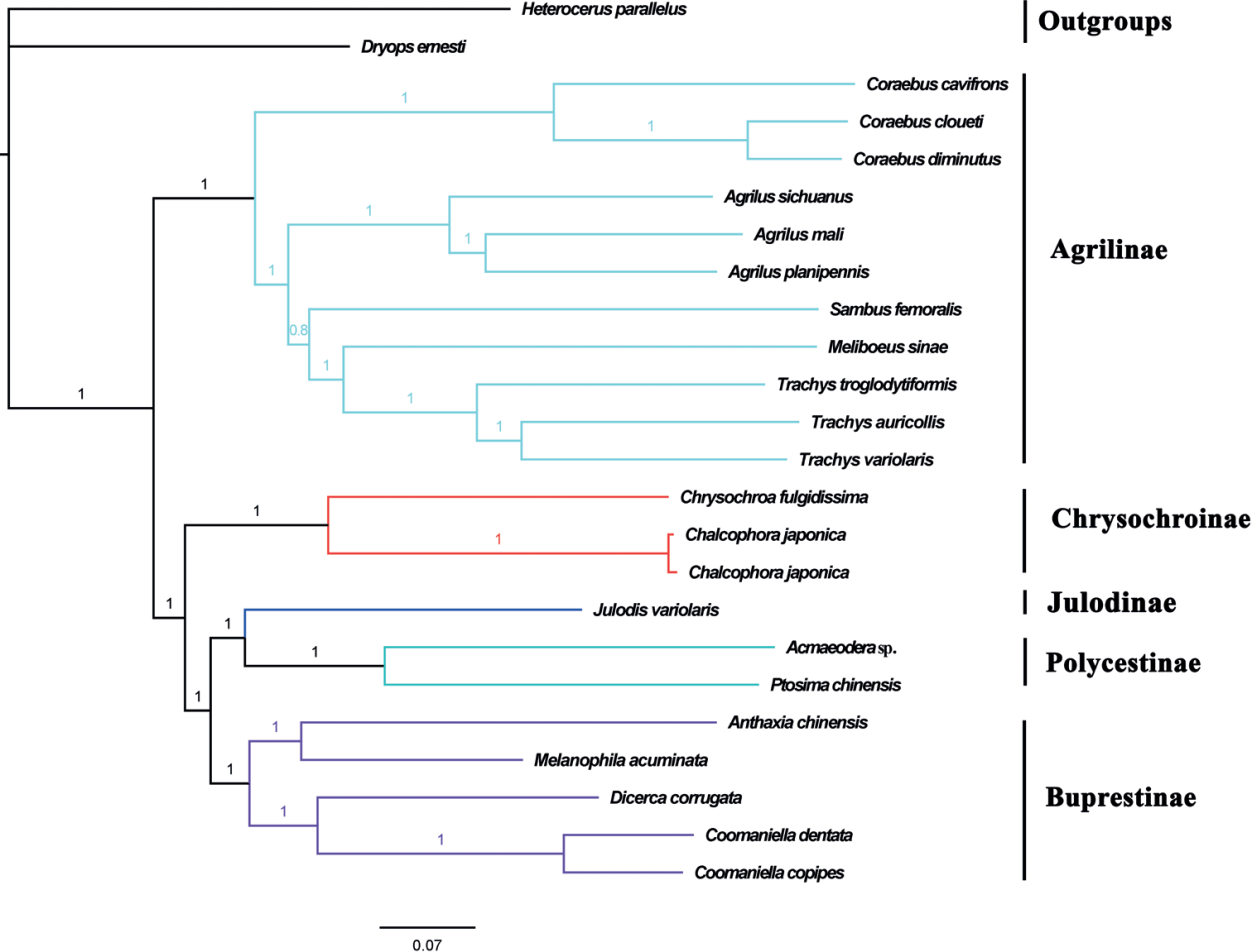
Phylogenetic relationships of studied species of Buprestidae using BI analyses based on 13 PCGs + 2 rRNAs of mitogenomes. The numbers on the branches show posterior probabilities.

For the concatenated sequences, the test of substitution saturation showed that the value of *I*_ss_ = 0.3910 was significantly smaller than *I*_ss.c_ = 0.8537 and *p* (0.0000) < 0.01, suggesting the sequences suitable for phylogenetic analysis. In the present study, both ML and BI trees using a nucleotide matrix (13 PCGs + 2 rRNAs) produced identical topologies (Fig. 5, Suppl. material 1: fig. S5), (Chrysochroniae + ((Julodinae + Polycestinae) + Buprestinae) + Agrilinae), in terms of subfamily-level relationship.

The target species *J.variolaris*, representing Julodinae, formed an independent clade close to Polycestinae with high support values (BI: 1; ML: 94), which supported the results of a previous study (Evans et al. 2015). The target species *P.chinensis* and *Acmaeodera* sp. are grouped together as an independent clade with high support values (BI: 1; ML: 100), representing Polycestinae. The Julodinae and Polycestinae formed a clade which was sister to Buprestinae with high support values (BI: 1; ML: 84). The target species *C.japonica* was clustered with other chrysochroine species as a clade, representing Chrysochroinae, with high support values (BI: 1; ML: 100). All the species of Agrilinae were clustered on one branch with high support values (BI: 1; ML: 100) and close to other buprestid clades, while the Coraebini was polyphyletic.

## ﻿Discussion

The gene composition and arrangement of these three mitogenomes are the same as other known buprestid mitogenomes (Cao and Wang 2019a, b; Xiao et al. 2019; Chen et al. 2021; Peng et al. 2021; Huang et al. 2022; Wei 2022; Weng et al. 2022). These three mitogenome had a positive AT skew, which was similar to most known buprestid mitogenomes (Duan et al. 2017; Cao and Wang 2019a, b; Xiao et al. 2019; Sun et al. 2020; Chen et al. 2021; Peng et al. 2021; Huang et al. 2022; Wei 2022; Weng et al. 2022). The genes *nad1* (*J.variolaris*, *P.chinensis*, and *C.japonica*), *nad4L*, and *nad5* (*C.japonica*) which started with TTG, GTG, and GTG, respectively, was also reported by previous studies in Buprestidae (Huang et al. 2022; Wei 2022). The Julodinae are closest to Polycestinae with high support values, which is consistent with the results of a previous study (Evans et al. 2015). The monophyly of Buprestidae has been corroborated once more, as all the buprestid species converge together as an independent clade (Evans et al. 2015; Huang et al. 2022; Wei 2022). In this study, the Coraebini was also found to be polyphyletic with the genera *Meliboeus* Deyrolle, 1864 and *Coraebus* Gory & Laporte, 1839 in different clades, also consistent with the previous studies (Evans et al. 2015; Huang et al. 2022; Wei 2022). Compared to Melanophilini, Coomaniellini is more closely related to Dicercini, which is in line with previous studies (Volkovitsh 2001; Evans et al. 2015; Huang et al. 2022).

In the present study, the sampling might be too limited to address the comprehensive phylogeny of Buprestidae. In the future, classification problems could be solved when enough mitogenomes are accumulated for more buprestid species, which requires the cooperation of taxonomists around the world.

## ﻿Conclusions

In this study, the complete mitogenomes of *Julodisvariolaris*, *Chalcophorajaponica*, and *Ptosimachinensis* were annotated and analyzed, of which the mitogenome of *J.variolaris* was the first complete mitogenome representative of the subfamily Julodinae. The three mitogenome sequences were of medium length (15,759–16,227 bp) in Buprestidae. These three mitogenomes shared the same gene order, which was consistent with those of known buprestid species. These three mitogenome sequences all had a high A + T content, and strong AT bias. All PCGs of the three species began with the typical ATN codon except *nad1* (*J.variolaris*, *P.chinensis*, and *C.japonica*), *nad4L* (*C.japonica*), and *nad5* (*C.japonica*) which were initiated with TTG, GTG, and GTG, respectively. In the present study, the BI and ML trees had exact same topologies with high-value support. The results of phylogenetic analyses also show that Julodinae is close to Polycestinae, the clade composed of Julodinae and Polycestinae is close to that of Buprestinae, and the Agrilinae clade is sister to that of (Chrysochroniae + ((Julodinae + Polycestinae) + Buprestinae)), and all the subfamilies are grouped in a monophyletic group with high support.

## Supplementary materials

Supplementary material 1 First mitochondrial genome of subfamily Julodinae (Coleoptera, Buprestidae) with its phylogenetic implications Data type table, images (word document) Explanation note Nucleotide composition of three newly generated mitogenomes. Circular maps of mitogenomes for *Julodisvariolaris*, *Ptosimachinensis*, and *Chalcophorajaponica*. The predicted secondary cloverleaf structure for the tRNAs of *Julodisvariolaris* (image S2), *Ptosimachinensis* (image S3) and *Chalcophorajaponica* (image S4). Phylogenetic relationships of Buprestidae using ML analyses based on 13 PCGs + 2 rRNAs of mitogenomes; the values one branches are bootstrap. https://binary.pensoft.net/file/795220This dataset is made available under the Open Database License (http://opendatacommons.org/licenses/odbl/1.0/). The Open Database License (ODbL) is a license agreement intended to allow users to freely share, modify, and use this Dataset while maintaining this same freedom for others, provided that the original source and author(s) are credited.Zhonghua Wei, Xuyan Huang, Aimin Shi
