# Supplementary material for: ﻿First mitochondrial genome of subfamily Julodinae (Coleoptera, Buprestidae) with its phylogenetic implications
Source: Zookeys. 2023 Jan 13;1139:165–82. doi: 10.3897/zookeys.1139.96216 (PMC9860506; doi:10.3897/zookeys.1139.96216)
Supplement: Supplementary material 1 — First mitochondrial genome of subfamily Julodinae (Coleoptera, Buprestidae) with its phylogenetic implications [file zookeys-1139-165_article-96216__-s001.docx]

**Supplementary material 1**

**Table S1.** Nudeotide composition of three newly generated mitogenomes.

| Taxa | Length (bp) | A% | C% | G% | T% | A+T% | G+C% | AT skew | GC skew |
| --- | --- | --- | --- | --- | --- | --- | --- | --- | --- |
| *Julodis variolaris* | 16,227 | 39.31 | 18.02 | 11.55 | 31.12 | 70.43 | 29.57 | 0.12 | -0.22 |
| *Ptosima chinensis* | 16,115 | 37.99 | 20.07 | 12.93 | 29.01 | 67.00 | 33.00 | 0.13 | -0.22 |
| *Chalcophora japonica* | 15,759 | 38.47 | 19.54 | 12.49 | 29.50 | 67.97 | 32.03 | 0.13 | -0.22 |


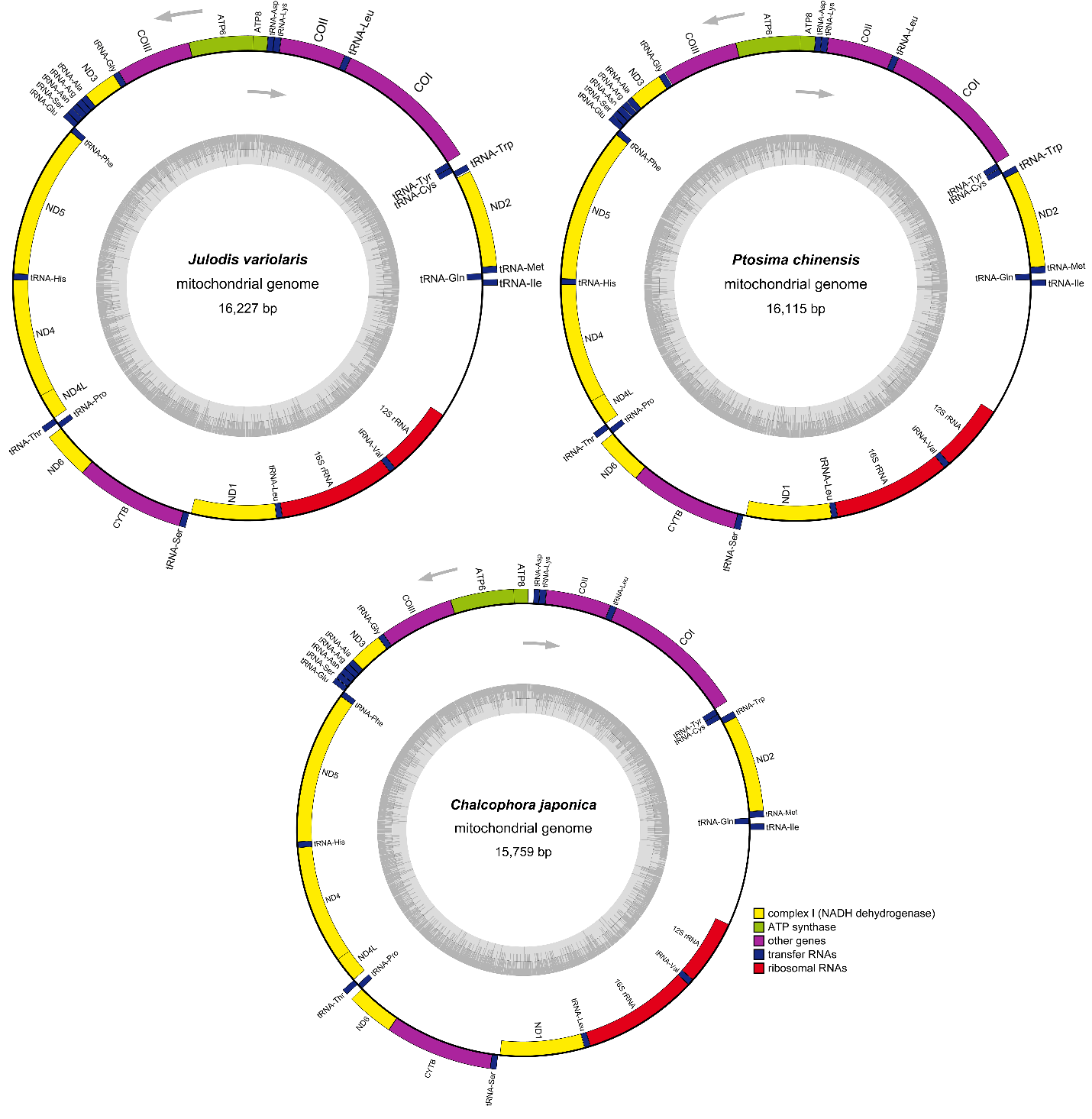


**Figure S1.** The Circular maps of mitogenomes for *Julodis variolaris*, *Ptosima chinensis* and *Chalcophora japonica*.

**
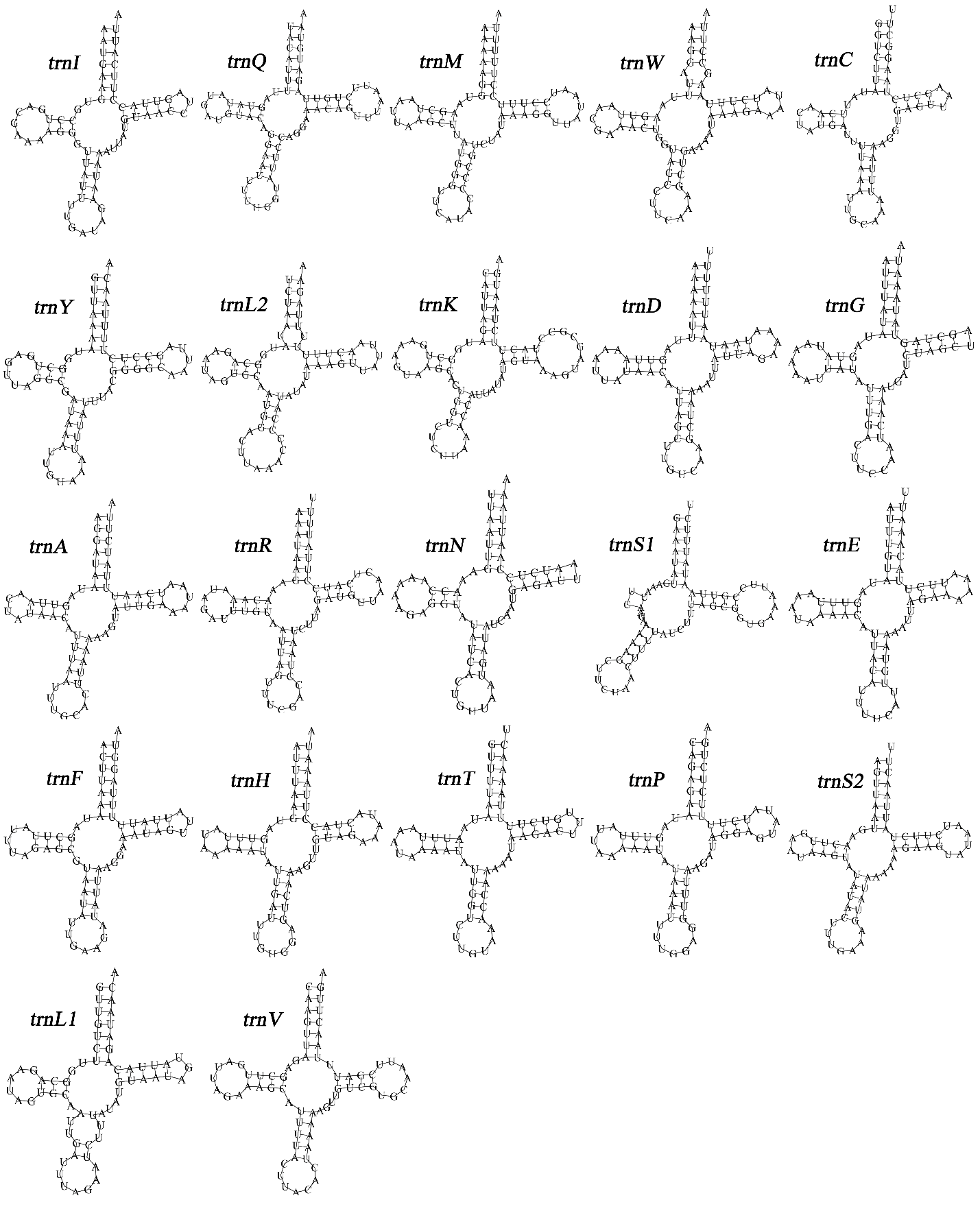
Figure S2.** The predicted secondary cloverleaf structure for the tRNAs of *Julodis variolaris*.

**
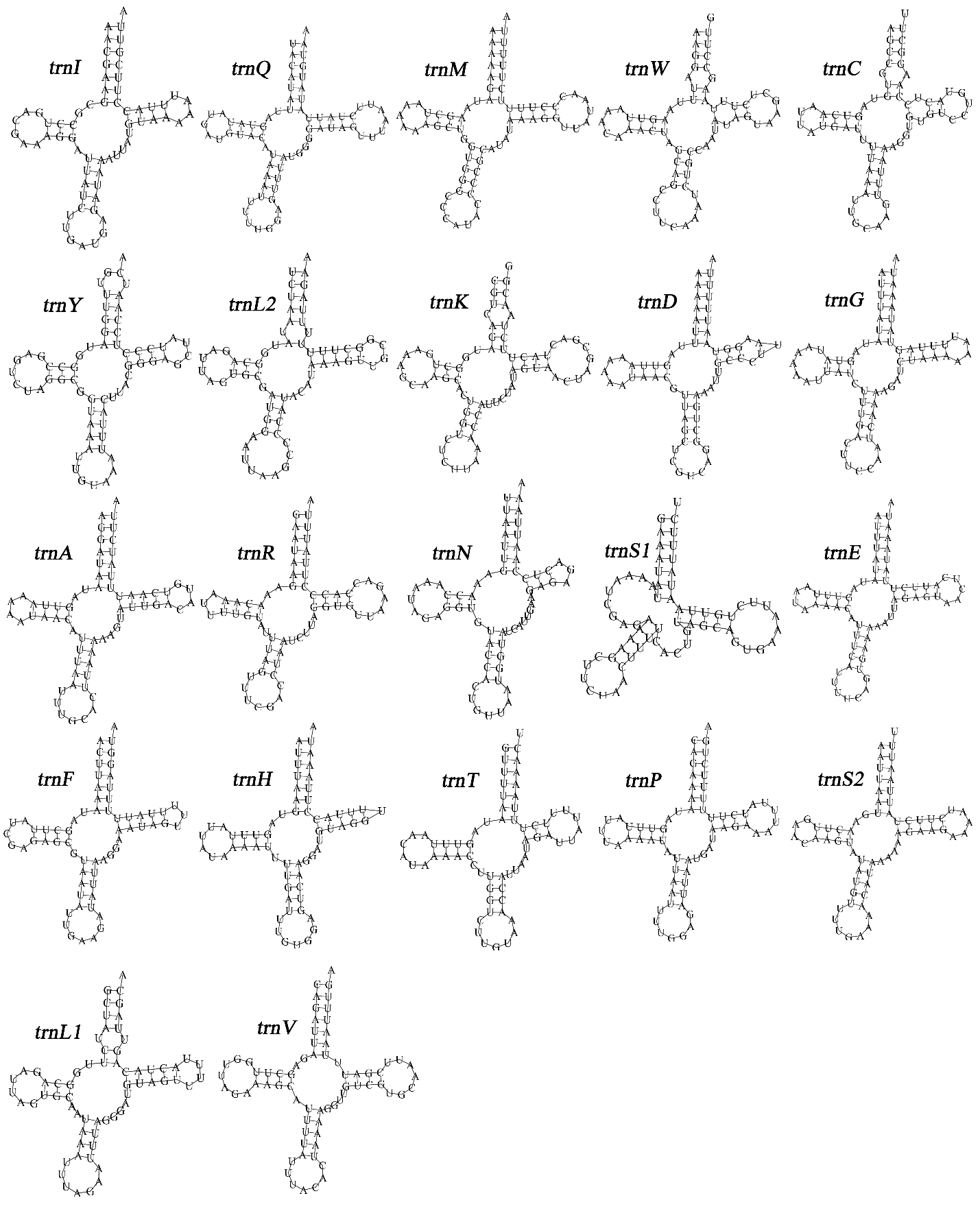
Figure S3.** The predicted secondary cloverleaf structure for the tRNAs of *Ptosima chinensis*.

**
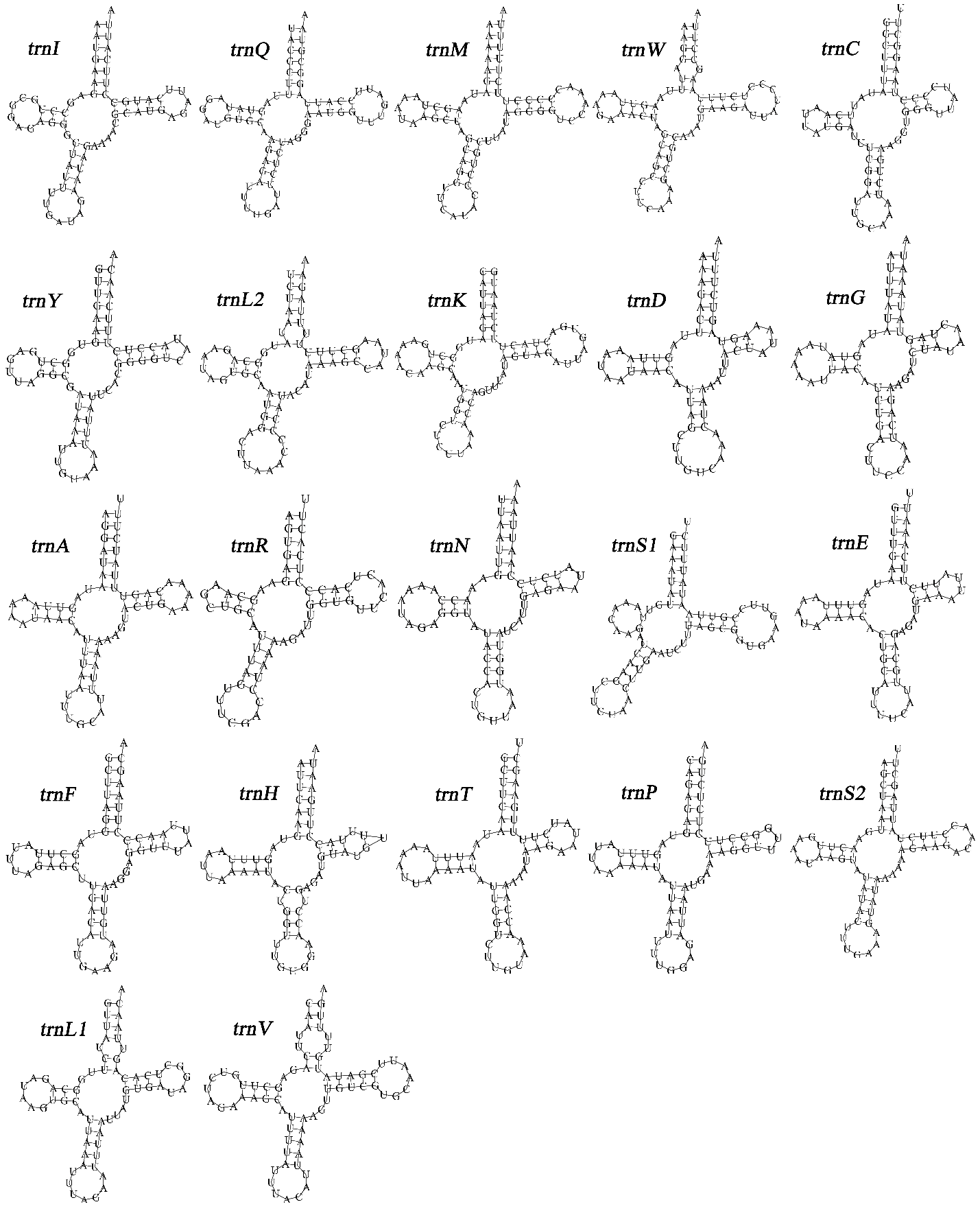
Figure S4.** The predicted secondary cloverleaf structure for the tRNAs of *Chalcophora japonica*.


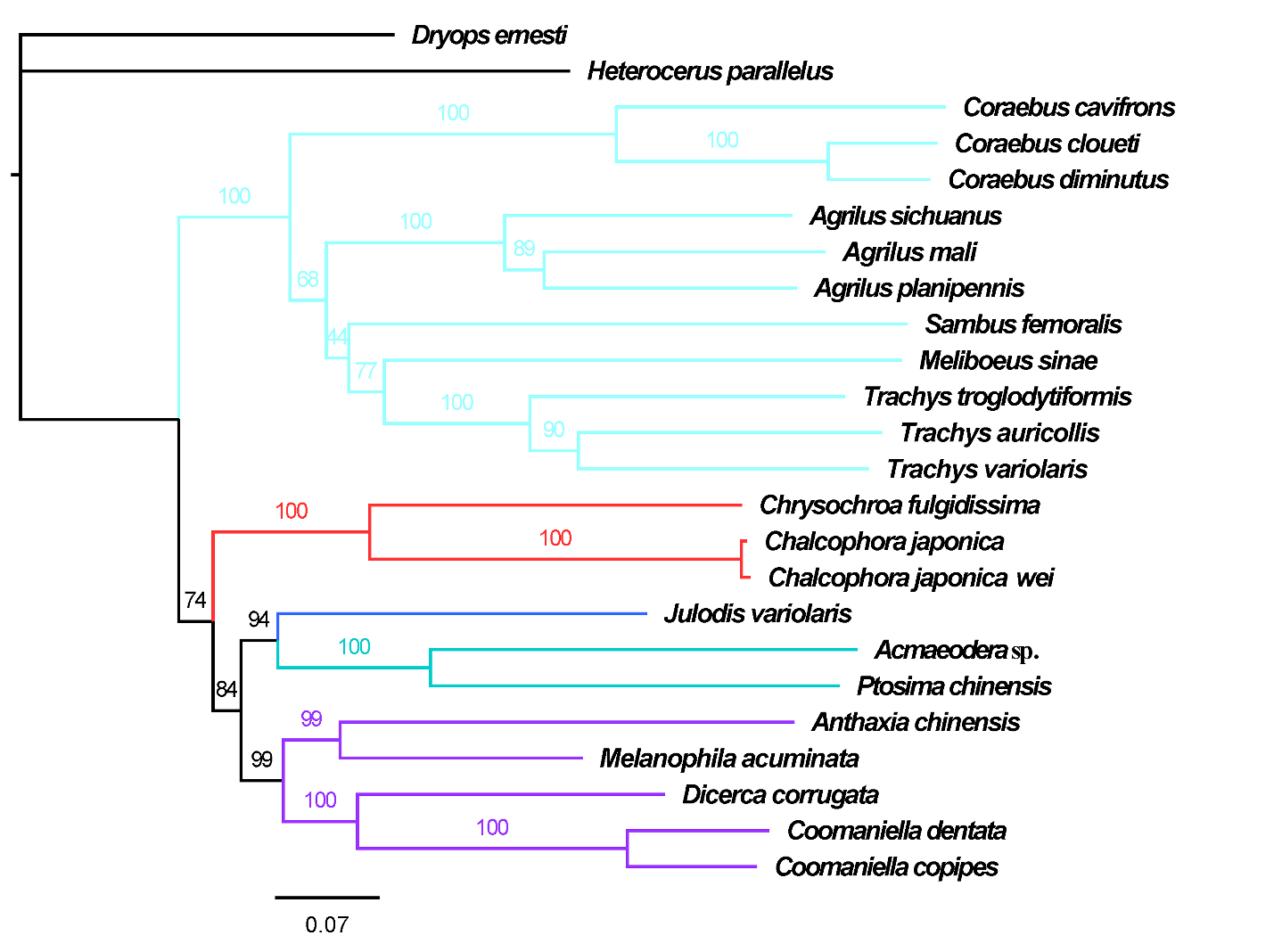


**Figure S5.** Phylogenetic relationships of Buprestidae using ML analyses based on 13 PCGs + 2 rRNAs of mitogenomes. The values one branches are bootstrap.
